# Genome-Wide Linkage Analysis and Association Study Identifies Loci for Polydactyly in Chickens

**DOI:** 10.1534/g3.114.011338

**Published:** 2014-04-21

**Authors:** Yanfa Sun, Ranran Liu, Guiping Zhao, Maiqing Zheng, Yan Sun, Xiaoqiong Yu, Peng Li, Jie Wen

**Affiliations:** * Institute of Animal Science, Chinese Academy of Agricultural Sciences, Beijing 100193, P.R. China; † College of Life Science, Longyan University, Longyan, Fujian 364012, P. R. China; § State Key Laboratory of Animal Nutrition, Beijing 100193, P. R. China; ‡ Fujian Provincial Key Laboratory of Preventive Veterinary Medicine and Biotechnology, Longyan, Fujian 364012, P. R. China

**Keywords:** chicken, polydactyly, linkage analysis, GWAS, candidate genes

## Abstract

Polydactyly occurs in some chicken breeds, but the molecular mechanism remains incompletely understood. Combined genome-wide linkage analysis and association study (GWAS) for chicken polydactyly helps identify loci or candidate genes for the trait and potentially provides further mechanistic understanding of this phenotype in chickens and perhaps other species. The linkage analysis and GWAS for polydactyly was conducted using an F2 population derived from Beijing-You chickens and commercial broilers. The results identified two QTLs through linkage analysis and seven single-nucleotide polymorphisms (SNPs) through GWAS, associated with the polydactyly trait. One QTL located at 35 cM on the GGA2 was significant at the 1% genome-wise level and another QTL at the 1% chromosome-wide significance level was detected at 39 cM on GGA19. A total of seven SNPs, four of 5% genome-wide significance (P < 2.98 × 10^−6^) and three of suggestive significance (5.96 × 10^−5^) were identified, including two SNPs (GGaluGA132178 and Gga_rs14135036) in the QTL on GGA2. Of the identified SNPs, the eight nearest genes were sonic hedgehog (*SHH*), limb region 1 homolog (mouse) (*LMBR1*), dipeptidyl-peptidase 6, transcript variant 3 (*DPP6*), thyroid-stimulating hormone, beta (*TSHB*), sal-like 4 (Drosophila) (*SALL4*), par-6 partitioning defective 6 homolog beta (*Caenorhabditis elegans*) (*PARD6B*), coenzyme Q5 (*COQ5*), and tyrosine 3-monooxygenase/tryptophan 5-monooxygenase activation protein, etapolypeptide (*YWHAH*). The GWAS supports earlier reports of the importance of *SHH* and *LMBR1* as regulating genes for polydactyly in chickens and other species, and identified others, most of which have not previously been associated with limb development. The genes and associated SNPs revealed here provide detailed information for further exploring the molecular and developmental mechanisms underlying polydactyly.

Polydactyly is a common limb malformation in human, mouse, chicken, and other vertebrates characterized by supernumerary digits. In chickens, where the usual number is four, polydactyly is the phenotype with a fifth digit on one or both feet (Warren 1944). Five digits is a breed characteristic in some chickens, including Beijing-You, Silkies, Dorking, Houdan, and Sultan. The chicken polydactyly locus was mapped to chromosome 2 (GGA2) through linkage analysis, in a region homologous to human Chr7q36 and mouse Chr5 (Pitel *et al.* 2000). This region is known to be important for human and mouse polydactyly traits and other limb malformation (Dundar *et al.* 2001; Dobbs *et al.* 2005; Lettice and Hill 2005). Huang *et al.* (2006) found that the T1254C polymorphism in exon 13 of *LMBR1* was strongly associated with polydactyly in chickens. Another study has shown complete association of this same single-nucleotide polymorphism (SNP; ss161109890) with the polydactyly phenotype in a manner that was concordant with the dominant and incompletely penetrant nature of this trait (Dorshorst *et al.* 2010). This study also searched the highly conserved zone of polarizing activity regulatory sequence (ZRS) for other SNPs that might explain this trait and found none. Several polydactylous breeds were compared, with the Silkie and Sultan breeds both having this SNP whereas three other breeds with polydactyly did not (Faverolle, Houdan, and Dorking). Due to the history of these breeds, the authors suggested there may be more than one allele or locus controlling polydactyly in the chicken, although in all of these breeds polydactyly still behaves as a single gene trait (Dorshorst *et al.* 2010).

The causality of the ss161109890 SNP located within the ZRS was subsequently demonstrated using *SHH* allelic imbalance and electroporation of mutant ZRS experiments. The ectopic expression of *SHH* in the anterior zone of polarizing activity is consistent with known causes of polydactyly in other species (Dunn *et al.* 2011; Maas *et al.* 2011).

The related genes for polydactyly were identified to better understand the molecular and developmental mechanisms underlying polydactyly. Genes related to hedgehog signaling, sonic hedgehog (*SHH*), and limb region 1 homolog (mouse) (*LMBR1*) are important regulatory genes for limb development (Lettice *et al.* 2012) and polydactyly traits in human, mouse, and chicken (Clark *et al.* 2001; Maas and Fallon 2004; Sagai *et al.* 2004, 2005; Huang *et al.* 2006; Albuisson *et al.* 2010; Dunn *et al.* 2011). Other candidate genes (*e.g.*, *EN2*, *HOXA10-13*, *GLI3*, *WNT2*, and *WNT16*) for the polydactyly trait were also found in different species (Gorbach *et al.* 2010). Quinn *et al.* (2012) identified a previously unrecognized role for *Zic3* in regulating limb digit number via its modifying effect on *GLI3* and *SHH* expression levels in mice. Kalsoom *et al.* 2013 have recently identified a novel zinc-finger gene (*ZNF141*) associated with autosomal-recessive postaxial polydactyly type A through whole-genome sequencing combined with homozygosity mapping and array comparative genomic hybridization analysis. These studies found the gene for one polydactyly allele/locus, but the molecular mechanism and other polydactyly alleles/loci remain incompletely understood (Robb and Delany 2012).

Both linkage analysis and genome-wide association studies (GWAS) can be used to map the quantitative trait loci (QTL) underlying complex traits. The GWAS, based on high-throughput SNP genotyping technologies, has revealed loci underlying a variety of interesting traits in domestic animals (Pearson and Manolio 2008) and it is more powerful than traditional linkage analysis in detecting genetic variants. In chickens, GWAS have been performed for growth and meat quality (Gu *et al.* 2011; Xie *et al.* 2012; Liu *et al.* 2013; Sun *et al.* 2013), egg production and quality (Liu *et al.* 2011), and other traits.

In the present study, both linkage analysis and GWAS were performed for the polydactyly trait using the Chinese Academy of Agricultural Science (CAAS) chicken F2 resource population derived from the cross between a local Chinese chicken breed (Beijing-You; BJY) and a commercial broiler line (Cobb-Vantress; CB) (Sun *et al.* 2013) to identify and provide information for allele/locus for polydactyly in chickens.

## Materials and Methods

### Animals and phenotypes

This research complied with the Guidelines for Experimental Animals established by the Ministry of Science and Technology (Beijing, China). The CAAS chicken F2 resource population was derived from a cross between BJY and CB chickens. The BJY is a Chinese indigenous breed with a 25−31% incidence of five digits on one or both feet. The CB (Cobb-Vantress) is a commercial broiler breed with four digits on each foot. Six BJY males were each mated to 12 CB females. Six males and 20 females of their offspring, the F1 generation, then produced the F2 chickens via artificial insemination and only nonrelated females to avoid inbreeding. The chickens from the three generations were raised in stair-step cages under the same routine conditions of nutrition and management at the conservation farm of the Institute of Animal Sciences (Sun *et al.* 2013).

A total of 400 chickens from three generation (including 367 F2 chickens from 20 full-sib families in five batches) were used. Blood was collected into acid citrate dextrose anticoagulant tubes from the brachial vein on d 56. The number of digits, four or five on one or both feet, was recorded. Two of six BJY males in the F0 generation had polydactyly. The pedigrees with polydactyly traits are shown in Supporting Information, Table S1.

### Genotyping and SNP quality

Genomic DNA was extracted from blood samples using the phenol-chloroform method. Genotyping was performed with 50 ng/µL genomic DNA using the Illumina 60K Chicken SNP Beadchip by DNA LandMarks Inc. (Saint-Jean-sur-Richelieur, PQ, Canada). A total of 39 were excluded due to sample call rate <90%. A total of 13,985 SNPs were removed for failing to meet one or more of the following conditions: SNP call rate <90%, minor allele frequency <3%, Hardy-Weinberg test P of < 10^-6^ and SNPs with no assigned chromosome or linkage group. After quality control measures, 42,585 SNPs distributed among 29 chromosomes (including the Z chromosome) and one linkage group (LGE22) remained (all SNPs information see File S1). The average physical distance between two neighboring SNPs was approximately 20.4 kb (Sun *et al.* 2013).

### Linkage analysis

The genetic linkage map was constructed using 19 full-sib families (six males and 12 females from F0, five males and 20 females from F1, and 148 males and 152 females from F2) and 42,585 SNPs with the improved version of CRI-MAP (2.503a, run in a 64-bit Unix system). This method has been described in previous studies (Groenen *et al.* 2009; Elferink *et al.* 2010). In genetic map construction, one full-family was excluded due to a computational problem.

To reduce the effect of linkage disequilibrium (LD) on the results, 6518 independent SNPs were acquired in all autosomal chromosomes using the indep-pairwise option, with a window size of 25 SNPs, a step of 5 SNPs, and r^2^ threshold of 0.2 (Sun *et al.* 2013). QTL analysis for the polydactyly trait used these same SNPs and web-based GridQTL software (Allen *et al.* 2012). A least-squares regression model was used for single QTL analysis, including the fixed effects of sex, hatch, and family, along with additive and dominance coefficients for the putative QTL. Significance thresholds were calculated using a permutation test (Churchill and Doerge 1994). A total of 10,000 permutations were computed to determine 5% and 1% chromosome-wide significance levels. 5% and 1% genome-wide significance levels were calculated following the Bonferroni correction:

$$P_{\mathrm{genome}\text{-}\mathrm{wide}}=1-{\left(1-P_{\mathrm{chromosome}\text{-}\mathrm{wide}}\right)}^{\mathrm{Ga}/\mathrm{Gc}}$$

Where Ga is the length genetic map of each chromosome and Gc is the length of the genetic map of all autosomal chromosomes. Confidence intervals for QTL positions were estimated by bootstrapping, as presented by Visscher *et al.* (1996).

### Statistical analysis for GWAS

The population structure was assessed by multidimensional scaling (MDS) analysis using PLINK software 1.07 (Purcell *et al.* 2007), as described previously in detail (Gu *et al.* 2011 and Sun *et al.* 2013). Pairwise identity-by-state distances were calculated between all individuals using the 6518 independent SNP markers, and MDS components were obtained using the mds-plot option based on the identity-by-state matrix.

The GWAS for polydactyly with 328 chickens from the F2 generation, and 42,585 SNPs were performed using the compressed mixed linear model (Zhang *et al.* 2010) and carried out using Tassel 3.0 software (Bradbury *et al.* 2007). The statistical model was:

$$Y_{ijklmn}=\mu_{i}+C1_{j}+S_{k}+B_{l}+G_{m}+K_{n}+e_{ijklmn},$$

where Y*_ijklmn_* are phenotypic values, µ*_i_* is the common mean, C1*_j_* is the effect of the first principal component, S*_k_* is the effect of sex, B*_l_* is the effect of batch of hatching (*l =* 1 – 5), G*_m_* is the effect of the SNP, K*_n_* is the random effect of the relative kinship matrix, which was constructed by matrix simple matching coefficients based on the independent SNPs, and this step was followed by compression (Zhang *et al.* 2010), and e*_ijklmn_* is the random residual effect.

The genome-wide significance P-value threshold was determined by the “LD adjusted’’ Bonferroni method (Duggal *et al.* 2008), which was calculated on the basis of the estimated number of independent markers and LD blocks in all chromosomes. The F2 population was estimated to have 16,760 “independent” tests, based on the “solid spine of LD” algorithm with a minimum D′ value of 0.8 calculated by Haploview 4.1 (Barrett *et al.* 2005). The two threshold P-values were established as 5.96 × 10^-5^ (1/16,760) for suggestive significance, and 2.98 × 10^-6^ (0.05/16,760) for 5% genome-wide significance. The Manhattan plot, showing genome-wide P-values of GWAS, was produced using R 2.13.2 software with the ‘‘gap’’ package (Zhao 2007).

## Results

### Linkage analysis

Before linkage analysis, the genetic linkage map was constructed using the CAAS chicken F2 population. A total of 42,585 SNP markers (each marker had 4−603 informative meiosis, average of 254), about 74% of all markers on the SNP beadchip, and 337 individuals from three generation and 19 full-sib families were used to construct the linkage map. As shown in Table 1, the total length of the sex-average map was 3040.8 cM, and there was no significant difference in total length between the male and female sex-specific maps (2929.2 and 2930.3 cM). The recombination rate of the map was 2.9 cM/Mb. The detailed information for the map is shown in Table S2.

**Table 1:** Linkage map lengths and recombination rates

Chromosome	Length, Mb*^a^*	Sex-Average, cM	Sex-Specific, cM		Recombination Rate, cM/Mb
			Female	Male	
GGA1	200.9	450.2	460.5	440.8	2.2
GGA2	154.8	308.4	328.7	288.5	2.0
GGA3	113.6	253.4	269.9	237.3	2.2
GGA4	94.2	199.9	211.2	188.7	2.1
GGA5	62.2	155.6	163.3	148.6	2.5
GGA6	37.3	102.7	110.2	95.3	2.8
GGA7	38.3	103.9	102.8	105.2	2.7
GGA8	30.6	90.9	92.5	89.4	3.0
GGA9	25.5	98.8	107.1	90.1	3.9
GGA10	22.5	82.2	86.2	78.3	3.7
GGA11	21.9	63.9	68.2	59.6	2.9
GGA12	20.5	75.1	79.5	70.7	3.7
GGA13	18.9	60.5	66.3	55.6	3.2
GGA14	15.8	67.8	71.3	64.4	4.3
GGA15	13	53.7	58.1	49.4	4.1
GGA16	0.4	0.0	0.0	0.0	−
GGA17	11.2	49.8	51.5	48.2	4.4
GGA18	10.9	48.0	48.7	47.4	4.4
GGA19	9.9	51.4	55.9	46.8	5.2
GGA20	13.9	54.3	56.1	52.5	3.9
GGA21	6.9	48.5	48.4	48.7	7.0
GGA22	3.9	51.6	52.0	51.3	13.2
GGA23	6	51.5	48.9	54.1	8.6
GGA24	6.4	47.9	44.3	51.5	7.5
GGA25	2	58.4	63.6	54.1	29.2
GGA26	5.1	48.3	47.5	49.2	9.5
GGA27	4.7	49.6	47.6	51.6	10.5
GGA28	4.5	48.7	53.1	44.4	10.8
LGE22	0.9	37.6	35.5	40.5	41.8
GGZ	74.6	228.3	-	228.3	−
Total autosomal	956.7	2812.5	2929.2	2702.0	2.9
Total length	1031.3	3040.8	2929.2	2930.3	2.9

a Physical length of the chromosome was based on the position of the last marker in the WASHUC2 build (2006).

A total of three hundred chickens from the F2 generation and 6518 independent SNPs (Table S3) in autosomal chromosomes were used in the linkage analysis for polydactyly. Quantitative trait loci locations and effects for the polydactyly trait are shown in Table 2. One QTL located at 35 cM (confidence interval from 23 to 63 cM) on GGA2 was significant at the 1% genome-wise level (Figure S1); it explained 9.34% of the phenotypic variation. Another QTL at the 1% chromosome-wide significance level was detected at 39 cM on GGA19 and it explained 5.35% of the phenotypic variation.

**Table 2:** Polydactyly trait loci mapped in the chicken CAAS F2 resource population

Location, cM/Mb	Confidence Interval, cM/Mb	F Ratios	A ± SE	D ± SE	PV (%)
GGA2 (35/0.8)	23.0−63.0/0.5−2.2	15.88**	0.17 ± 0.03	0.05 ± 0.04	9.34
GGA19 (39/0.5)	22.0−45.0/0.3−0.7	9.06*	0.03 ± 0.03	0.18 ± 0.06	5.35

CAAS, Chinese Academy of Agricultural Science; A, additive; D, dominance; PV, percent of phenotypic variance explained by the QTL; PV%, [(MSR − MSF)/MSR] × 100 where MSR is residual mean square in the reduced model and MSF is the residual mean square in the full model; QTL, quantitative trait loci.

* 1% chromosome-wide significance.

** 1% genome-wide significance.

### GWAS results

After 39 samples were excluded because of low sample call rate and one lost phenotypic record, 327 chickens (286 with four digits, and 41 with five digits) from the F2 generation were used for further analysis.

The MDS analysis of the 16,760 SNPs using the first two MDS components showed that the chickens within each full-sib family were clustered together (Sun *et al.* 2013). To correct for population stratification, the first MDS component was used as a covariate in the model as suggested (Price *et al.* 2006; Gu *et al.* 2011; Sun *et al.* 2013).

**Figure 1:**
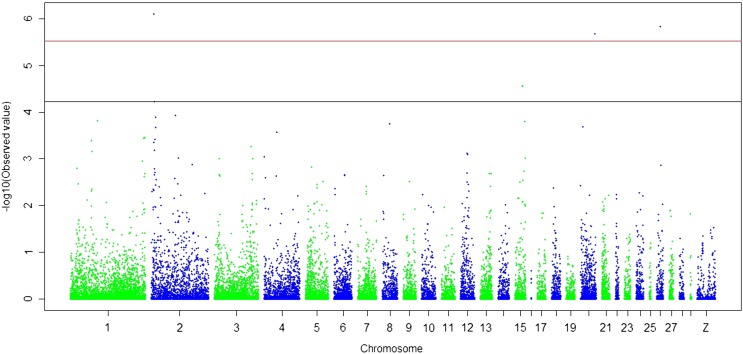
Manhattan plot showing the association of all single-nucleotide polymorphisms with the polydactyly trait. Single-nucleotide polymorphisms are plotted on the x-axis according to their position on each chromosome against association with these traits on the y-axis (shown as -log10 P-value). The black line indicates genome-wise significance of suggestive association (P = 5.96 × 10^−5^), and the red line indicates genome-wise 5% significance with a P-value threshold of 2.98 × 10^−6^.

As shown in Figure 1 and Table 3, four SNPs associated with polydactyly were of genome-wide significance, and three SNPs had suggestive significance. These SNPs explained 6−9% of the phenotypic variation.

**Table 3:** Significant SNPs associated with chicken polydactyly

Chromosome	SNPs	Position, bp	P Value*a*	P Adjust*b*	MAF	R*b*	Nearest Gene
2	GGaluGA132178	6641213	7.94E-07	0.01	C/A-0.10	0.09	*DPP6* D 9.5 kb
2	Gga_rs14135036	7628179	5.96E-05	1.00	A/G-0.31	0.06	*SHH* D 113.9 kb; *LMBR1* U 240.7 kb
15	Gga_rs15023766	9240112	2.74E-05	0.46	C/A-0.17	0.07	*COQ5* U 22.9 kb
15	Gga_rs14093104	8755174	2.74E-05	0.46	G/A-0.17	0.07	*YWHAH* U 2.8 kb
20	GGaluGA181512	12887215	2.08E-06	0.03	C/A-0.19	0.09	*SALL4* U 176.3 kb
20	Gga_rs16175635	12900524	2.08E-06	0.03	A/G-0.19	0.09	*PARD6B* D 176.4 kb
26	Gga_rs13606164	2579119	1.47E-06	0.02	A/G-0.14	0.09	*TSHB* U 35.6 kb

SNP, single-nucleotide polymorphism; MAF, minor allele frequency of the first allele; R^2^, the proportion of phenotypic variation explained by the SNP; U, the SNP is upstream of the gene and D is downstream.

a P value from mixed linear model models.

b LD-adjusted Bonferroni P value.

The most significant SNP, GGaluGA132178, was located on GGA2 at 6641.2 kb (P = 7.94 × 10^-7^), 9.5 kb downstream of dipeptidyl-peptidase 6, transcript variant 3 (*DPP6*). Another SNP, Gga_rs14135036, located on GGA2 at 7628.2 kb was suggestively associated with polydactyly (P = 5.96 × 10^-5^). This SNP was in close proximity to other candidate genes for polydactyly, 113.9 kb downstream of sonic hedgehog (*SSH*) and 240.7 kb upstream of limb region 1 homolog (mouse) (*LMBR1*).

The SNP Gga_rs13606164, located at 2579.1 kb on GGA26, is 35.6 kb downstream of the thyroid stimulating hormone, beta (*TSHB*) gene, had highly significant association with the polydactyly (P < 2.98 × 10^-6^). Two other SNPs (GGaluGA181512 and Gga_rs16175635), located within a 13.3-kb segment on GGA20, were of genome-wide significance (P < 2.98 × 10^-6^); one was 176.3 kb upstream of the Sal-like 4 (*Drosophila*) (*SALL4*) gene and the other was 176.4 kb from the Par-6 partitioning defective 6 homolog beta (*C. elegans*), (*PARD6B*) gene.

Two SNPs (Gga_rs15023766 and Gga_rs14093104) with suggestive association (both p-values were 2.74 × 10^−5^) with polydactyly were located within a 484.99-kb segment on GGA15; one was 22.9 kb downstream of coenzyme Q5 (*COQ5*), and the other was 2.8 kb upstream of tyrosine 3-monooxygenase/tryptophan 5-monooxygenase activation protein, etapolypeptide, (*YWHAH*).

## Discussion

In the present study, the QTLs and candidate genes for polydactyly were identified in chickens by the use of both linkage analysis and GWAS. A high-density genetic linkage map is the basis for linkage analysis. The current map is comprised of 29 chromosomes and one linkage group, with a total size of 3040.8 cM. The map size was similar with the high-resolution chicken genetic map using 13340 SNPs and 1619 chickens (Elferink *et al.* 2010). The present map ensures accuracy and precision of linkage analysis. In the present study, two QTLs (one located at 35 cM on GGA2 and another located at 39 cM on GGA19) were identified through linkage analysis. The QTL on GGA2 was also found in previous studies in chicken (Dunn *et al.* 2011; Yang *et al.* 2013) and other species.

For GWAS analysis, the compressed mixed linear model was one of the most effective methods for controlling false-positive results in GWAS (Huang *et al.* 2010; Zhang *et al.* 2010) and ensured reliability of the results. The population-based GWAS has more power than traditional family-based linkage analysis (Klein 2007) and it can detect more SNPs of interest that are in or near potential candidate genes. The GWAS results obtained here were in accord with the linkage analysis on GGA2 and obtained more detail of relevant SNPs. The GWAS also found additional potential loci on other chromosomes. Seven significant SNPs were identified near the potential candidate genes or the important regulatory genes for polydactyly. The genes, *SHH* and *LMBR1* on GGA2, already known to be important for polydactyly in chickens and other species, were identified again here. Variations of *LMBR1* were found to influence digits numbers in chickens (Huang *et al.* 2006; Hang *et al.* 2007). Both *LMBR1* and *SHH*, were shown to be essential for normal development of the chicken limb (Lettice *et al.* 2003; Lettice and Hill 2005; Dunn *et al.* 2011). The *SHH* and *LMBR1* were also in the QTL region identified here by linkage analysis. Another gene *DPP6*, near the QTL peak in GGA2, was also identified here. Mutations of *DPP6* produce human disease and cause movement disorders (Tanaka *et al.* 2011).

The gene *SALL4* encodes a putative zinc finger transcription factor and plays a role in modulating embryonic stem cell pluripotency and early embryonic development (Zhang *et al.* 2006). Variations of *SALL4* are associated with upper limb abnormality in humans (Kohlhase *et al.* 2002; Paradisi and Arias 2007). Given what is known of the roles of this gene in limb development, the present GWAS findings suggest that it is a potential candidate gene for polydactyly in chickens.

Four other genes, *TSHB*, *PARD6B*, *COQ5*, and *YWHAH*, are involved in growth and development, cell differentiation, or signal transduction (Dibrov *et al.* 1997; Phillips 2004; Nolan *et al.* 2008; Grover *et al.* 2009; Alarcon 2010), but there has been no suggestion that they are involved in limb development. The present results indicate that they might be potential candidate genes for polydactyly and so they are deserving of further study in this regard.

In conclusion, two QTLs and eight genes (*SHH*, *LMBR1*, *DPP6*, *TSHB*, *SALL4*, *PARD6B*, *COQ5*, and *YWHAH*) were identified through combined linkage analysis and GWAS for the polydactyly phenotype in chickens. In addition to confirming earlier studies in chickens and other species that *SHH* and *LMBR1* are important regulating genes for polydactyly, the other genes identified here provide additional information for potentially exposing the molecular mechanisms and developmental biology underlying polydactyly in chickens. As new candidate genes, especially the *SALL* gene, they will be targets for future work.

## Supplementary Material

Supporting Information
