# Supplementary material for: Genome-Wide Linkage Analysis and Association Study Identifies Loci for Polydactyly in Chickens
Source: G3 (Bethesda). 2014 Apr 21;4(6):1167–72. doi: 10.1534/g3.114.011338 (PMC4065260; doi:10.1534/g3.114.011338)
Supplement: Supporting Information [file supp_4_6_1167__index.html]

Genome-Wide Linkage Analysis and Association Study Identifies Loci for Polydactyly in Chickens — Supporting Information 

# Genome-Wide Linkage Analysis and Association Study Identifies Loci for Polydactyly in Chickens

## Supporting Information for Sun *et al.*, 2014

**Files in this Data Supplement:**

- Supporting Information - Figure S1, File S1, and Tables S1-S3 (PDF, 128 KB)
- Figure S1 - Polydactyly QTL analysis on GGA2 in the CAAS chicken F2 population. (PDF, 108 KB)
- File S1 - Genotype (.xlsx, 50 MB)
- Table S1 - The pedigrees with polydactyly traits in the CAAS chicken F2 population. (.xlsx, 18 KB)
- Table S2 - Genetic map of the CAAS chicken F2 population. (.xlsx, 2 MB)
- Table S3 - Independent SNPs used in linkage analysis and multidimensional scaling analysis. (.xlsx, 187 KB)
