# Supplementary material for: Genome-Wide Linkage Analysis and Association Study Identifies Loci for Polydactyly in Chickens
Source: G3 (Bethesda). 2014 Apr 21;4(6):1167–72. doi: 10.1534/g3.114.011338 (PMC4065260; doi:10.1534/g3.114.011338)
Supplement: Supporting Information [file supp_g3.114.011338_011338SI.pdf]

G3 · Genes | Genomes | Genetics

Genome-wide Linkage Analysis and Association Study Identifies Loci for Polydactyly in Chickens

Yanfa Sun*†#[1], Ranran Liu*§[1], Guiping Zhao*§, Maiqing Zheng*§, Yan Sun*, Xiaoqiong Yu*, Peng Li*, Jie Wen*§[2]

*Institute of Animal Science, Chinese Academy of Agricultural Sciences, Beijing 100193, P.R. China; †College of Life Science, Longyan University, Longyan, Fujian 364012, P.R. China; §State Key Laboratory of Animal Nutrition, Beijing 100193, P.R. China; #Fujian Provincial Key Laboratory of Preventive Veterinary Medicine and Biotechnology, Longyan, Fujian 364012, P.R. China

[1]Yanfa Sun and Ranran Liu contributed equally to this work.
[2]Corresponding author
*§Yuanmingyuan West Road No. 2, Haidian District, Beijing 100193, P.R. China
†#Dongxiao North Road No. 1, Xinluo District, Longyan, Fujian 364012, P.R. China

DOI: 10.1534/g3.114.011338

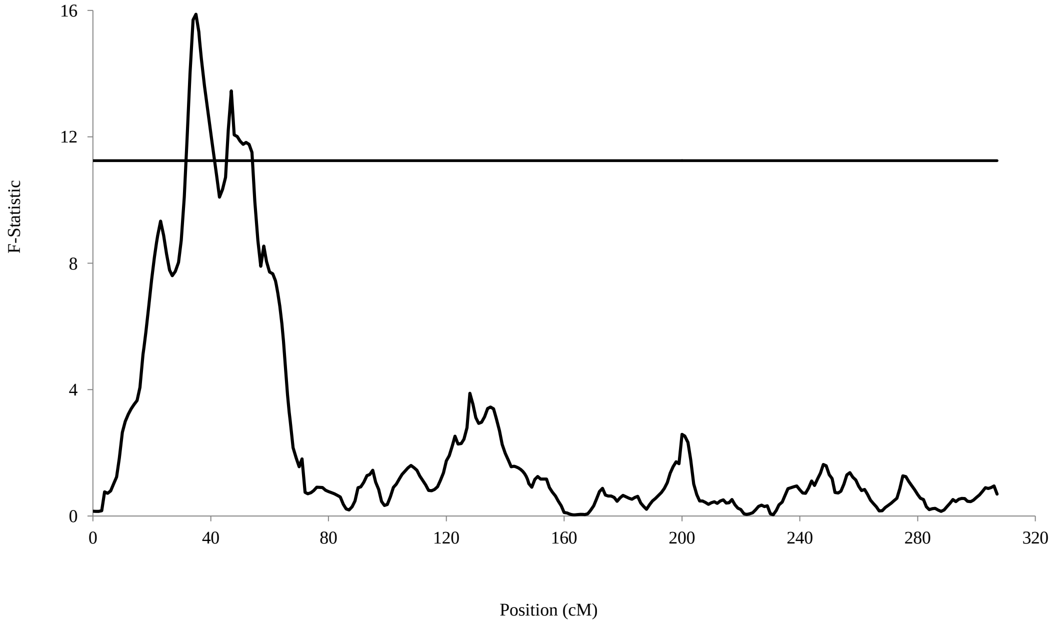

Figure S1 Polydactyly QTL analysis on GGA2 in the CAAS chicken F2 population. The bold line shows the 1% genome-wide significance threshold.

File S1

Genotype

Available for download as an Excel file at http://www.g3journal.org/lookup/suppl/doi:10.1534/g3.114.011338/-/DC1

Tables S1-S3
Available for download as Excel files at http://www.g3journal.org/lookup/suppl/doi:10.1534/g3.114.011338/-/DC1

Table S1 The pedigrees with polydactyly traits in the CAAS chicken F2 population.

Table S2 Genetic map of the CAAS chicken F2 population.

Table S3 Independent SNPs used in linkage analysis and multidimensional scaling analysis.
