# Supplementary material for: Genome-Wide Linkage Analysis and Association Study Identifies Loci for Polydactyly in Chickens
Source: G3 (Bethesda). 2014 Apr 21;4(6):1167–72. doi: 10.1534/g3.114.011338 (PMC4065260; doi:10.1534/g3.114.011338)
Supplement: Supporting Information [file supp_g3.114.011338_FigureS1.pdf]

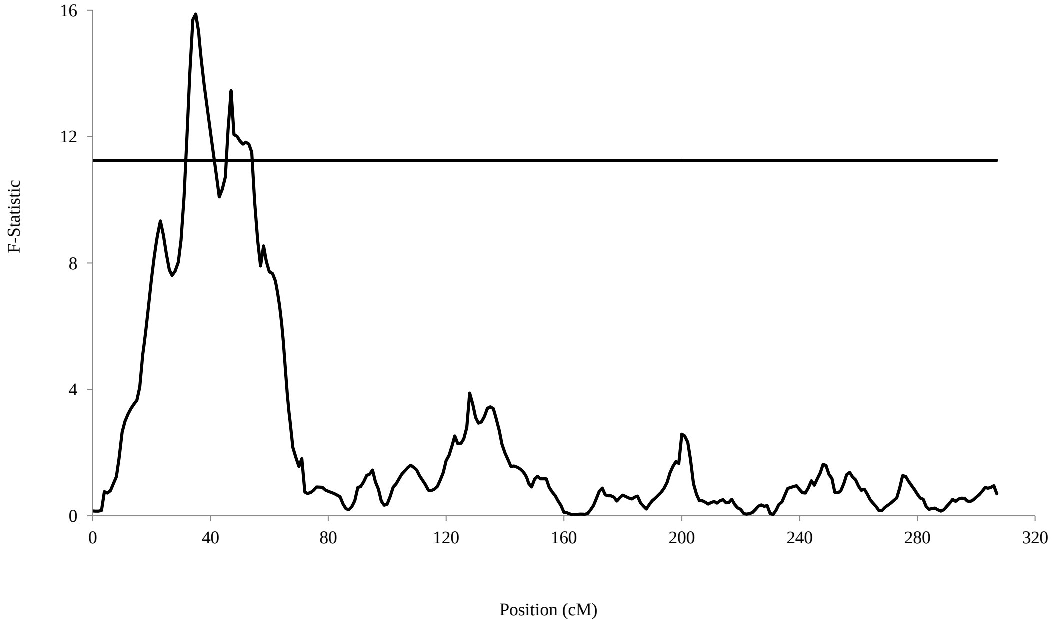

Figure S1 Polydactyly QTL analysis on GGA2 in the CAAS chicken F2 population. The bold line shows the 1% genome-wide significance threshold.
